# Limitations and Pitfalls of Using Family Letters to Communicate Genetic Risk: a Qualitative Study with Patients and Healthcare Professionals

**DOI:** 10.1007/s10897-017-0164-x

**Published:** 2017-11-01

**Authors:** Sandi Dheensa, Anneke Lucassen, Angela Fenwick

**Affiliations:** 1 Clinical Ethics and Law, Southampton General Hospital, South Academic Block, University of Southampton, Room AB 203, MP 801, Tremona Road, Southampton, SO16 6YD, 02381 205082 UK; 2 Wessex Clinical Genetics Service, University Hospital Southampton NHS Foundation Trust, Southampton, UK

**Keywords:** Family communication, Genomics, Inherited genetic conditions

## Abstract

European genetic testing guidelines recommend that healthcare professionals (HCPs) discuss the familial implications of any test with a patient and offer written material to help them share the information with family members. Giving patients these “family letters” to alert any relatives of their risk has become part of standard practice and has gone relatively unquestioned over the years. Communication with at-risk relatives will become an increasingly pressing issue as mainstream and routine practice incorporates broad genome tests and as the number of findings potentially relevant to relatives increases. This study therefore explores problems around the use of family letters to communicate about genetic risk. We conducted 16 focus groups with 80 HCPs, and 35 interviews with patients, recruited from across the UK. Data were analyzed thematically and we constructed four themes: 1) HCPs writing family letters: how to write them and why?, 2) Patients’ issues with handing out family letters, 3) Dissemination becomes an uncontrolled form of communication, and 4) When the relative has the letter, is the patient’s and HCP’s duty discharged? We conclude by suggesting alternative and supplementary methods of communication, for example through digital tools, and propose that in comparison to communication by family letter, direct contact by HCPs might be a more appropriate and successful option.

## Introduction

**Fig. 1:**
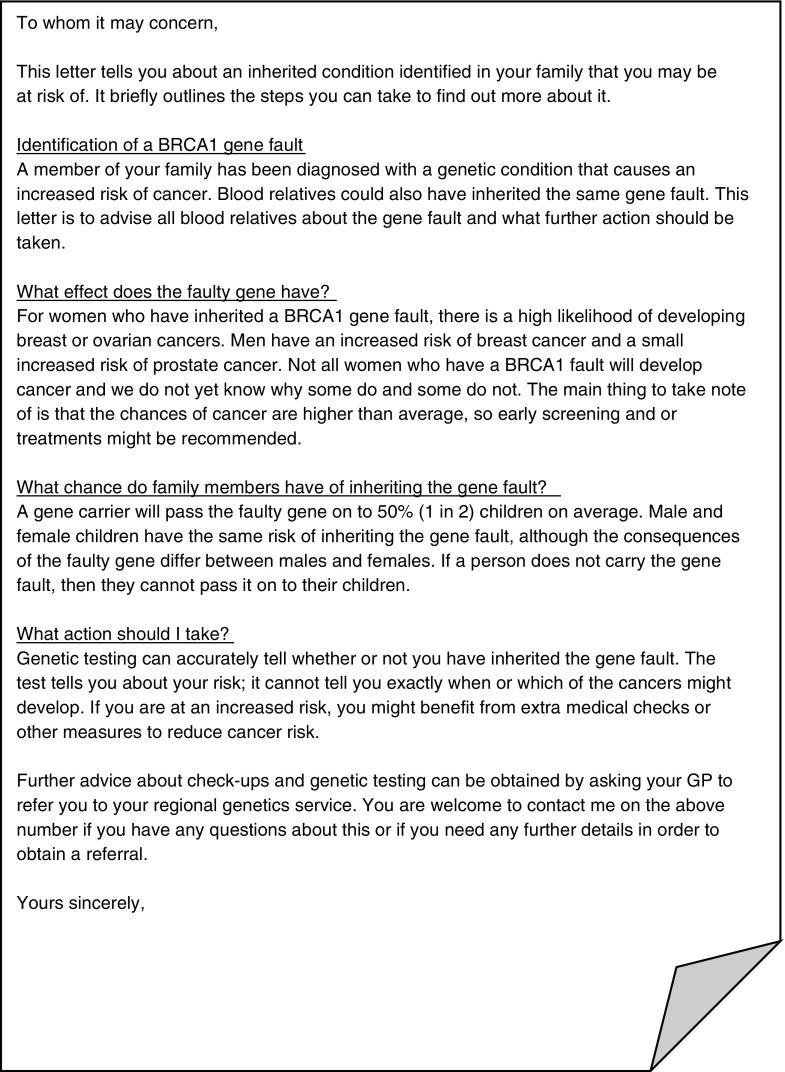
Example of a letter

Patients diagnosed with an inherited risk generally perceive that they have a responsibility to tell their at-risk relatives (Chivers-Seymour et al. 2010; Dheensa et al. 2016; Hallowell et al. 2003). Healthcare professionals (HCPs) similarly feel a responsibility to ensure these relatives are made aware of their risk (Dheensa et al. 2015). European genetic testing guidelines recommend that HCPs discuss the familial implications of any test with a patient, as well as a strategy on how to tell relatives. They advise genetic counselors to offer the patients “written material to help the counselee to spread the information in the family” (Kääriäinen et al. 2009). Offering letters, often called family letters, to patients to give to their relatives is a common way for HCPs to facilitate communication and help to ensure that those at risk gain appropriate information to allow them to make informed choices about whether to seek advice about having a genetic test themselves (Mendes et al. 2016; Ormondroyd et al. 2014; Wilson and Etchegary 2010). Neither European nor British Society for Genetic Medicine guidelines suggests a standard or a template for a family letter (Joint Committee on Medical Genetics 2011). In general, letters inform the recipients about the familial inheritance and encourage relatives to seek a referral to discuss testing. Figure 1 shows an example of a letter.

Although European guidelines mention written material, the way that family letters are used in different countries is unclear. UK genetic HCPs often write generic letters without specifying to the patient who the recipients should be, meaning this is left for patients to work out. In the Netherlands, genetic HCPs specify the relatives to whom patients should give letters (I van Langen, personal communication, Aug 2017). In a Dutch study about the dissemination of these letters, 56 cardiogenetics patients were asked to distribute 249 letters and the number of at-risk relatives referred to the clinical geneticist and/or cardiologist within two years was fairly high at 57% (142 of 249) (van der Roest et al. 2009). This suggests that the use of letters can be somewhat effective.

However, a number of studies about family communication have shown that patients do not always tell their relatives about a risk, and that this is for a range of reasons. These include feeling a desire to protect oneself and family from potentially harmful information and/or having a dysfunctional relationship with the relative, lack of contact or closeness with them, or poor understanding about the risk and its relevance to others (Chivers-Seymour et al. 2010; Mendes et al. 2017; Wiens et al. 2013). Thus, other ways for HCPs to help to ensure that relatives become aware of their risk have been suggested in the literature. For example, De Geus et al. (2016) have tested the feasibility of an intervention that uses motivational interviewing principles to improving hereditary cancer counselees’ knowledge about which at-risk relatives to inform and what information to disclose. Several other intervention studies have offered additional genetic counseling or family therapy about family communication for a range of conditions, and these have significantly increased the number of relatives told about their risk (Eisler et al. 2016; Forrest et al. 2008; Hodgson et al. 2016). For example, Hodgson et al. (2016) found, through a randomized controlled trial, that communication of risk to relatives was higher for patients who were given a specifically designed telephone counseling intervention. In a different study, Eisler et al. (2016) delivered an intervention based on family therapy principles to help families cope with and talk about their genetic condition with family members. They trained genetic counselors to run multi-family discussion groups in which different families could learn from and support each other. The researchers found that families thought the intervention valuable, acceptable and feasible. A limitation is that some genetic services might be unable to incorporate these specific interventions due to constrained resources and, for example, a lack of access to family therapy expertise (Eiser et al. 2016). Thus, HCPs might rely upon family letters. Interestingly, in the final stage of an intervention by Forrest et al. (2008), 8/11 index patients had not yet told their relatives about the risk. The researchers offered these eight participants a family letter and the option for a HCP to hand it out for them. Only three participants accepted a letter and all three opted to hand it out themselves. The paper gives no further detail about the letters.

Several other intervention studies have focused on the use of letters more specifically and have shown that when HCPs send letters directly to relatives, testing uptake significantly increases (Evans et al. 2009; Suthers et al. 2006). In families with familial breast and ovarian cancer, hereditary non-polyposis colorectal cancer, or Cowden syndrome, Suthers et al. (2006) found that in usual practice (i.e. patients giving out letters), an average of 23% of relatives in each family had a genetic test after two years, compared with 40% in the intervention group (HCPs sending relatives letters directly). Evans et al. (2009), who contacted relatives about BRCA1/2 mutations, found that uptake after direct contact by HCPs was especially higher among men. Another study about hereditary colon cancer showed that direct contact by HCPs led to testing uptake that was comparable to contact by patients (Aktan-Collan et al. 2007).

Although some genetic services feel obliged to contact at-risk relatives directly to alert them of their risk (van der Roest et al. 2009), a qualitative study about UK clinical genetics practice showed that only a minority of HCPs would consider making direct contact with the relative (or their GP) even when a patient had specifically asked them to (Dheensa et al. 2017). HCPs preferred to rely on patients to pass on the information for various reasons: doing so arguably enhanced their patient’s autonomy (patients get to decide when and how exactly to pass on the message). HCPs furthermore argued that relying on patients to communicate would be better for their own relationship with their patient, and their patients’ relationship with their family members. Specifically, they worried that contacting relatives could amount to “interfering” in family relationships, which could cause or exacerbate any existing family tensions. Moreover, they considered patients to be better placed than themselves to tell relatives because they would often have a relationship with them, so would know how best to communicate with them and how they might react. HCPs additionally argued that it would be too resource-intensive to do anything other than give their patient these letters. In cases where the patient was refusing to tell their relatives, or where it was unclear whether the patient had told them, HCPs worried that direct contact would breach patient confidentiality.

For these reasons, the practice of giving family letters to patients has gone relatively unquestioned over the years. In fact, recently there have been suggestions for, and shifts towards, expanding the use of letters. For example, Baars et al. (2016) recently proposed giving letters to counselees with a negative BRCA result as a means of giving them some support. Furthermore, a recent case of non-disclosure of a BRCA risk in a family in the Netherlands has placed more pressure on HCPs there to ensure relatives are aware of their risk, often via these letters (Vrijenhoek 2015). Finally, in France, legislation has been passed that obliges HCPs to give their patients these letters (D’Audiffret Van Haecke and de Montgolfier 2016). Yet studies show that communication often fails (Dheensa et al. 2015). This suggests relying on patient-mediated letters needs investigation.

It is a particularly timely point to explore the use of family letters as the primary means of ensuring communication. Worldwide, projects such as the UK 100,000 genomes project, are underway, which aim to incorporate whole-genome approaches into clinical practice (Caulfield 2016). Such testing is starting to be offered outside the genetics clinic, by HCPs for whom communication with family members is new territory (Caulfield 2016). Results from genome tests, at least initially, will make communication with family members even more important: HCPs will often need to test family members to determine the significance of genetic findings or to contact at-risk relatives about so-called incidental findings in families where there is no typical family history of the condition (Shkedi-Rafid et al. 2014). These questions are timely also because cases where communication about genetic risk has failed to happen have reached UK courts, raising issues about HCPs’ responsibilities to relatives (Chico 2016; Fay 2016; Gilbar and Foster 2016; Mitchell et al. 2016).

### Purpose of the Study

This paper aims to explore problems around the use of family letters to communicate about genetic risk. To give our study focus, we examined communication between adults, firstly because communication with children would not happen by letter, and because a body of literature has already explored the unique issues raised when parents communicate risk information to children (e.g., Eisler et al. 2016; Metcalfe et al. 2011; Plumridge et al. 2011; Rowland and Metcalfe 2013). Originally, the focus groups and interviews were organized as part of a study that aimed to explore patients and HCPs’ views and experiences of consent, confidentiality, and sharing information in genetic medicine. Some of the specific research questions included whether HCPs and patients consider genetic information as confidential at the individual or familial level and whether they perceive HCPs to have a responsibility to patients’ relatives (Dheensa et al. 2015, 2016, 2017). Much of the resultant data provided novel insight into familial communication, including the use of letters.

## Methods

### Recruitment and Sample

There were two participant groups: HCPs and “patients”: people who have had a genetic test and/or are affected by the signs, symptoms, and routine surveillance associated with a condition. One exception was P34 whose spouse had died from a Lynch syndrome cancer, whose children were at risk, and who requested an interview.

We invited HCPs who order genetic tests to take part through presentations at professional meetings and by sending emails with attached information sheets to heads of departments for dissemination to colleagues. Sampling was purposive: the aim was to recruit from a range of genetic and affiliated services. Since HCPs work and make decisions in teams, we chose cross-sectional (one-off) focus groups instead of interviews, with groups working in the same department where possible, to provide an understanding of the real-life context in which HCPs worked (Vaughn et al. 2006). We held 16 focus groups with 80 HCPs from across the UK, either in clinical departments or during professional meetings. Table 1 contains details of their professions. We have a relatively high number of focus groups because in the UK there are 23 genetic centers, as well as one cancer center that conducts genetic testing routinely. In the original study, we wanted to gain a picture of local practices in as many centers as possible—our HCP participants came from 14 of the 24 centers. It is worth noting here that there were no striking differences between centers regarding the use of letters.

**Table 1:** Professions represented across the focus groups

Profession	*n*
Genetic counselors	37
Clinical scientists (molecular/cytogenetics)	16
Consultants in clinical genetics	8
Registrars (trainees) in clinical genetics	8
Nurses working with a genetics team	4
Fetal medicine professionals	4
Family history coordinators	2
Nephrologist	1
Total	80

Of the 16 professional focus groups (FGs) in this study, FGs 5, 6 and 7 comprised of genetic counselors who worked in different genetic centers. In all other FGs, all participants worked in the same team as each other. FG2 and FG9 comprised of clinical laboratory scientists (molecular or cytogeneticists)

Local collaborators helped to recruit patients via three large UK genetics centers (who posted information about the study onwards to all recent patients seen for conditions amenable to intervention, such as hereditary cancers and cardiac conditions, in the previous two years) We also posted study information on social media sites for specific conditions and for genetic conditions generally. Although one “patient” participant had a Huntington disease test, we focused on conditions conventionally thought of as amenable to medical intervention (e.g. familial cancers and cardiac conditions where there is treatment, risk-reducing option, or surveillance), since here, communication with at-risk relatives is most urgent. Since patients lived in different parts of the country, had different conditions, and had potentially sensitive experiences to discuss, we offered interviews rather than participation in focus groups. SD (the first author, a social scientist with expertise in the ethical and social aspects of genetics/genomics) contacted those who sent back an expression of interest slip or responded online to arrange a suitable time and date and, for face-to-face interviews, a suitable location. There were 35 adult participants from England (one of whom was also a HCP in FG14). Table 2 contains details of patient participants’ genders, conditions, test results, and issues they faced regarding communication about the risk.

**Table 2:** Demographic characteristics of patient participants (*N* = 35)

Variable	Number of participants
Gender	24 women (W); 11 men (M)
Test status	31/35 had tested positive for their condition. Of the remaining five: − 2/35 had tested negative (P8,cardiomyopathy,W and P11,Huntington,W) − 2/35 had not yet had a test but were undergoing surveillance (P5, possible Lynch,W and P12,HBOC,W) − 1/35 was the partner of patient who died of a Lynch syndrome related cancer and had at -risk children (P34,Lynch,W)
Condition/risk	Hereditary breast/ovarian cancer (HBOC): 14/35 (5/14 had a diagnostic test after cancer) Lynch syndrome: 9/35 (7/9 had diagnostic test after a cancer) Familial Adenomatous Polyposis (FAP): 3/35 (1/3 had a diagnostic test after a cancer) Alport syndrome: 4/35 Hereditary cardiomyopathy: 2/35 Hereditary haemochromatosis: 1/35 Huntington disease: 1/35 Undisclosed: 1/35 (HCP who was also a patient)
Learning about risk	Most learned of their risk at the same time as siblings and other close relatives. 7/35 were probands and told family about their risk immediately, often before having a test/getting their result
Disclosure	None had withheld information although a few had not told distant relatives yet, mostly because they had no contact details. Three participants’ relatives did not share information about risk with them. P5 (possible Lynch,W): cousin was withholding his exact mutation so she could not have a definitive test. P18 (HBOC,W): sister did not want to tell her about risk directly so asked her General Practitioner (GP) to do so. P18 found out months later as GP failed to pass the message on. P30 (HBOC,W): sister did not share her HBOC diagnosis. P30 found out because a nurse mentioned it during an appointment with the affected sister, which a third sister attended. The third sister then told P30 about the risk.

### Data Collection

SD conducted all audio recorded focus groups/interviews, which lasted approximately one hour. Since focus groups comprised on average just five participants, we did not consider it necessary to involve an observer—SD fulfilled what would be an observer’s role (e.g., taking field notes of non-verbal communication, such as nodding). A limitation of this approach is that some observational data might have been missed (Stewart and Shamdasani 2014). The interview schedule and topic guide (provided in Table 3) were adapted over time to pursue emerging lines of inquiry. We ceased recruitment and data collection as we approached saturation: when the main categories had depth and variation (Corbin and Strauss 2015). Data were generated between late 2013 and early 2015.

**Table 3:** Topic guide and interview schedule

Group	Questions
HCPs	• Introductory questions (e.g., what is your role? What kinds of patients do you see? How many per week?) • Is confidentiality important in the area of medicine that you are working in? • What aspects of the medical consultation should be kept confidential? • Are there guidance documents or protocols you follow for confidentiality? • How, if at all, do you talk about the limits of confidentiality in the consent process? • Regarding genetic test results, who do you think should be the one to tell the result to at-risk relatives? • Have you ever had experience of a patient telling you they were not going to inform their family of risk? Or a patient who you weren’t sure had told? • To what extent do you feel like you have a responsibility to make sure patients’ family members know they are at risk? • Regarding these issues, do you feel like you have enough support and training? • Who do you talk to about ethical issues? • What are your main ethical concerns, if you have any? • Do you have some other things you would like to raise?
Patients	• Introductory questions (e.g., tell me about yourself) • Can you start by telling me why you first thought about having a genetic test? • How did you feel before you went to talk to the genetics team? • What made you decide to have the test/not to have the test? • (Where relevant) Can you tell me what it was like receiving your results? • What do you think happens to your test result? • What does privacy/confidentiality/secrecy mean to you? • Whose responsibility is it to tell at-risk family members of risk? • What are your hopes and concerns about genetic testing? • Do you have some things you would like to add?

### Data Analysis

To analyze data, we drew on a thematic analysis method (Braun and Clarke 2006). All three authors (SD, AF, a social scientist with expertise in medical ethics, and AL, a consultant in clinical genetics with expertise in the ethical aspects of genetics/genomics) participated in coding and constructing themes. We initially analyzed the datasets (HCPs and patients) separately. Analysis involved repeatedly reading transcripts, coding data, and creating themes. More specifically, we went through the data line by line, labelled salient features with codes, and identified key questions and potential themes. To combine the data from the two groups, we used an adapted version of the “following a thread” method, which is used in mixed-methods studies: we selected certain codes and potential themes (“threads”) from one dataset and investigated the way they were discussed in the other (Moran-Ellis et al. 2006; O’Cathain et al. 2010). The other dataset raised additional questions and ideas about the code or potential theme, which we then explored further by going back to the first dataset. We thus moved iteratively, back-and-forth, between the two datasets, which allowed us to identify areas of convergence and complementarity, as well as divergence. During this process, we built up our codes into themes and then reviewed the themes to ensure they accurately reflected the codes. Ongoing analysis helped us to refine the themes and the overall narrative of our data. All authors critically reviewed the thematic story to improve the rigor—specifically the credibility and confirmability—of the analysis.

## Results

The resultant themes reflect each step involved in communication by letter: 1) HCPs writing family letters: how to write them and why?, 2) Patients’ issues with handing out family letters, 3) Dissemination becomes an uncontrolled form of communication, and 4) When the relative has the letter, is the patient’s and HCP’s duty discharged? In the quotes below, patients and professionals’ names are replaced with identification codes (e.g., focus group 1 participant 1 = FG1P1, patient 1 = P1). We also indicate the patients’ condition and gender (with M for man and W for woman).

### Theme 1: HCPs Writing Family Letters: How to Write Them and Why?

HCPs struggled with how to word family letters: whether it was appropriate to use wording that told at-risk relatives, directly, to seek a test, or whether to leave this decision up to them. That is, they worried about how directive they should be, especially as they could not know which relatives the patient would disseminate the letter to (and in turn, whether testing could actually be helpful for the recipient). HCPs also worried that letters might frighten recipients:

FG1P4: Our letters are quite gentle, “We’ve seen a family member and some people might be at risk, if you want to find out if you’re one of those people, get in touch”. [Colleague] received a letter from another genetics service, that said, “You’re probably aware of the gene mutation that’s been found in your family, if not, this is probably going to be really distressing for you to read.”

FG1P5: That was the first paragraph…

FG1P4: That was the opening paragraph. As long as you’re not giving someone a letter that says, “This is terrible news.”

FG1P5: “This is a death sentence.”

While a letter that told relatives they were at risk might make them too frightened to seek a referral, one that was vague might not lead them to seek a referral at all:

FG14P1: The wording can have quite an influence on whether someone does then act on it.

FG14P4: You don’t write anything to scare the living daylights out of them. You write a gentle letter…It’s difficult to know how forceful to be, “You must go and be seen.”

FG14P1: It’s trying to strike a balance between not frightening someone, but making them aware that it’s important information that could be relevant to them and change things.

FG14P4: But it’s hard because you’ve never met this person and you don’t know how they’re going to respond to the letter.

FG14P3: Yeah, everyone will read it differently. Some people will interpret it as, “That’s something I could do,” and others as, “This is a thing I must do.”

HCPs also pointed out that the reading age of the letters they wrote was probably too high [FG4P1] and so the information would likely need further explanation at a clinic appointment. These kinds of considerations were important to HCPs because once they identified an inherited tendency in their patient, they felt responsible for telling at-risk relatives who might also have inherited the same condition. Using a letter was their way of acting on this responsibility. As the quotations above suggest, they acknowledged that the letter was not an optimal way to communicate, but they did identify some benefits of using them. For example, they thought that the letter would at least “make it easier” for patients to communicate with relatives because that having written information would boost their confidence, which could in turn make it more likely that they would talk to relatives about the risk:

FG7P3: I use letters a lot, “Pass these on to your relatives.” I’m quite surprised at the difference in confidence that people have. They feel they’ve got some backup. People are scared of getting information wrong when they pass it on. It’s about that making it easy to share information. I think that’s where we can really focus our efforts.

Patients echoed this and told their relatives to read the letter for an explanation of the risk;

P17: I said [to my children], “If you read the letter, it’ll explain it.” (M, Lynch)

### Theme 2: Patients’ Issues with Handing out Family Letters

While HCPs thought the letter would be helpful for patients, patients did not always agree. They thought the letter was more helpful for their GPs:

P34: I didn’t think that there was any…the only reason behind that letter was just the ticket to get your GP to do something for you. (W,Lynch)

Other patients saw the letter as having symbolic value; once they had been given it, they felt pressured to tell their relatives about the risk. Importantly, they also felt they were obliged, where possible, to have a conversation with their relatives or send them an accompanying letter or email of their own. This obligation extended to relatives with whom they had no, or a poor, relationship. They felt uncomfortable about giving relatives the letter unexpectedly as this could cause the recipient to become distressed or anxious:

P26: If I hadn’t told them and they suddenly got something out of the blue through the post, they would have wondered why I hadn’t said anything…there are some who obviously may find it very difficult. (W,BRCA)

One HCP corroborated these patients’ views, i.e., receiving a letter could be upsetting: FG5P1 reported having received a “*series of angry phone calls*” from relatives of a patient who had posted the letters, without explanation, enclosed in Christmas cards.

HCPs recognized that for patients, having this conversation with relatives, or writing them a personalized letter or email, took “*a huge amount of emotional energy and thought, time, and care*” [FG8P1]. Indeed, patient participants reported that planning their strategy for communication with at-risk relatives and dealing with the consequences of communication was extremely difficult, as one participant—who was both a patient and a HCP member of FG14—highlighted:

P35: The logistics of doing that and the problems it can cause. It’s ended up in people not speaking in the family, and then you’re left, as the person disseminating that letter, with that. I’ve decided to give the letter to my brothers, and I know all my nephews have not been told. And I love my nephews. I am put now in a very difficult position. (W,undisclosed condition)

While many of these are issues relevant to communicating genetic findings within families in general, the findings showed that the use of *letters* was not helpful for solving problems, and in fact, using them could create more problems*.* For example, the above quotation highlights how patients handing out letters could end up becoming complicit in keeping family secrets because gatekeepers in the family could block the letter and further communication. Since handing out the letters and talking to at-risk relatives was so emotionally distressing, one patient argued that it “*shouldn’t be up to*” her and thought HCPs should have told her relatives instead:

P9: It was horrible circumstances, a funeral. I’ve got a younger sister, but we don’t know who her dad is [and whether she’s at risk]. I didn’t want her to find out through a letter, and I haven’t spoken to her for years, so I was put in this predicament where I had to speak to her. I was angry. I’ve got enough to deal with, and I’m having to worry about telling everybody in my family, and it shouldn’t be up to me. I could be dead by the age of 40, and yet I’m expected to deliver this, deliver that, find this address, go through this person, go through…the arguments it caused….(W,FAP)

HCPs knew that patients were communicating in these difficult circumstances. They pointed out another difficulty: that at-risk relatives might “overreact” to the news of a risk, leaving patients to deal with the additional responsibility of trying to “educate” them. HCPs remarked that the prospect of this led some patients not to talk to relatives at all:

FG6P3: Some people don’t want to share because it’s going to be blown out of all proportion, and for some quite treatable conditions—some bleeding conditions, there is treatment, it can be managed well—yet they don’t want to share that with their family. They say it’s going to be turned into something that it’s not, which you hear a lot, or I hear a lot, in clinic.

Patients were thus triply burdened: burdened with their own diagnosis, with the emotional and practical effort of communicating, and with causing and managing arguments and secrets. As the quotation below illustrates, any notion that patients might “*like*” the responsibility of disseminating information (e.g., because it would endow a sense of control) was quickly countered in the focus groups, because HCPs knew that communication was onerous:

FG8P5: Do you think people like that responsibility [of sending letters] though? Or do you think they actually feel then they have to be the messenger and get shot?

FG8P1: I think it’s a real burden for some of them, absolutely.

FG8P5: I sometimes worry a bit about that.

FG8P3: The people that they don’t know or haven’t seen for ages, then they suddenly have this party or wedding and then they go distributing these letters. Sometimes it’s the only chance they’ll have.

FG8P4: It’s not ideal.

FG8P2: Often at funerals.

Despite this, many HCPs did not think that sending letters directly to at-risk relatives was an appropriate part of their role, for a range of reasons such as worrying about the ethical aspects, and social implications, of approaching people to whom they had no duty of care. For example, they worried about not knowing the intricacies of family dynamics and that direct contact might worsen any existing conflicts:

FG13P2: If a patient’s not prepared to communicate with family members, there’s already a dynamic issue there. I don’t want to be responsible for potentially making that worse.

Another participant worried that a letter from a HCP might place influence on a relative to have a test:

FG2P3: The clinical geneticists, I don’t think they’re allowed, they can’t go out and tout for business.

### Theme 3: Dissemination Becomes an Uncontrolled Form of Communication

HCPs highlighted two examples of how the letter could became an uncontrolled form of communication. The first example was that some patients passed letters on too widely—to relatives at no risk or minimal risk. Patients might do this due to a misunderstanding or due to taking a “better safe than sorry” approach. Notably, HCPs said that sometimes, these relatives were referred in to see them and wanted a test. HCPs said they felt conflicted about whether they ought to test them, given their potentially low risk and that testing them could reveal the result of an intervening relative. HCPs tried to obviate these issues by specifying the relatives that patients needed to tell. However, patients’ faulty interpretations of risk were difficult to undo:

FG14P8: [Whether and to whom they pass on the letter] can be [based on] so many sets of objectives and thoughts about what they’ve had in the family. It might be, therefore, if they disseminate it within the family, they still just disseminate with [the idea that] “it’s always been the blokes”, “always been the girls”, and so on, and it’s really hard to get rid of that, I think.

A second example came from FG8, in which a mother took some letters from her daughter without permission. Here, the loss of control manifested in the patient not being in control of whom to tell and when:

FG8P1: I had a patient with a BRCA mutation. Her mum hadn’t had testing: we didn’t know whether it had come down mum’s side. The mum took it upon on herself to let everybody know, to hand out letters at the exit of the church at a funeral or some family gathering. So, my patient is furious: she felt that was her information to give and [that it was] her right to choose when she gave that information. She felt a lot of guilt that it had started with her. It’s created real family conflict.

This HCP pointed out that the patient’s mother’s actions can be seen as particularly undermining of the daughter when considering the time and care that patients usually put into telling relatives about the risk.

### Theme 4: When the Relative Has the Letter, Is the Patient’s and HCP’s Duty Discharged?

Like proband patients, participants who had received family letters themselves talked about how having a letter created a sense of obligation. In this case, the obligation was to seek testing, if only to help their relatives. A quote from P22, who had female grandchildren, illustrates this point:

P22 I was informed by this letter that my sister gave to me. It was a formal introduction to faulty BRCA gene. So then, I thought about it and I decided I had to take the test. I couldn’t ignore the fact that this process had been initiated. Having had the letter, I had to take the test, in my view. I couldn’t ignore it, for the sake of potentially other people in the family …Having been informed about it transmit[ted] a pressure to take the test. (M,BRCA)

Some participants who were probands said that unlike P22, their relatives did not seek a referral upon reading the letter. Some even declined the letter: the relatives were apparently anxious and they coped with this anxiety through avoidance:

P9 I said to [cousin], “There’s a chance it’s in the family; do you want one of the letters?”. He said, “I don’t want to know; I don’t need to know. I went to the doctors a couple of weeks ago and I had a full MOT [check-up] and there’s nothing wrong with me.” The same with [another cousin], “Oh, I think I’d know by now if I was ill.” I went, “Okay, you can’t say I haven’t told you.” (W,FAP)

This finding echoes HCPs’ worries: that letters worded too strongly could frighten relatives, but being unspecific about the risk might lead to apathy towards testing:

FG8P2: Some of the “to whom” letters are quite, not vague, but you don’t want to give them risk figures, because you don’t want to scare people. So, you keep them quite simple.

As P9’s quotation above hints at, patients felt that once they had talked to their relatives about the risk and handed on the letter, to an extent, they had discharged their responsibility. They had done all they could, having alerted them and talked to them about the risk and referred them to the letter for more information. They could not, and did not see it appropriate to, “talk their relatives into” having the test, and the letter certainly did not convince the relatives to do so:

P17: I said “If you read the letter it’ll explain it, and it is wise that you get checked yourself.” My lad, he, he don’t even talk. I asked my daughter, “Has he said anything?” I got “No.” So I just, I don’t honestly think he’ll have it done.

I: Have you tried to convince him or do you think you need to?

P17: No, I’ve said to him, “Well, I think you should have it done, simply because you’ll be checked.” (M,Lynch)

Avoidance might be easier or more likely if a letter is sent without any accompanying discussion, but patients’ experiences indicated that even after such conversations, family members may not take up the offer of testing. It is not clear from these findings what the nature of the conversations were and whether patients might have contributed to their relatives’ anxiety.

HCPs reflected that there was an over-reliance on letters, and patients needed open channels through which they could contact HCPs for help with communication:

FG8P1: We give the letters, and we trust that most families will pass it on, but we don’t routinely check who’s been told and who hasn’t. We rely on them sharing that.

FG8P2: We rely on them [the patients].

HCPs worried that being handed letters symbolized some “finality” to patients—that the clinical encounter was over—and thought there ought to be some way to remedy this:

FG14P8: No-one’s ever got back to me to say “I can’t bring myself to [pass on letters].”

FG14P5: But there isn’t a pathway for that person to know they can come back [to us].

FG14P1: I don’t think the patients get back to us nearly as often as they could, or should. I think they just keep quiet and lie low.

Some participants in this focus group wondered whether there ought to be a “*family communication officer*” to see patients six months after their diagnosis. P35, the patient-HCP from FG14, agreed that options for follow up would be helpful:

P35 It would have been nice to have had somebody to come back to. Because I was left. I would have liked somebody to have shared that burden, and to have said, “They’re not listening, I don’t know what to do, I’ve got this knowledge, help me.” (W,undisclosed condition).

## Discussion

This study has explored some of the reasoning around, and problems with, using family letters to communicate genetic risk to at-risk relatives. Since the use of such letters has become a standard practice where inherited tendencies are identified, and since they are likely to be used more as we enter the genomic age, our exploration is timely. We found that the letter had several practical and symbolic functions. These included boosting patients’ confidence when talking about the risk to relatives; alerting a GP to the need for a referral to a genetic service; placing pressure on probands to tell relatives; placing pressure on relatives to have a genetic test; and as a tool to help HCPs and patients discharge their moral duty to relatives.

We found that there were difficulties at each step in the process of using the letters. For example, when writing them, HCPs worried about striking the balance between encouraging relatives to have a test while indicating they had a choice about having one, and worried about how to word letters in a way that was clear but would not cause alarm. This finding echoes previous research about genetic counseling summary letters: compared with shorter letters, specially written by medical writers, patients found letters written by HCPs too long and complicated. They caused anxiety and lower perceived confidence around understanding their condition (Brown et al. 2016; Roggenbuck et al. 2015).

When giving out letters, patients felt obliged to make contact and talk with their relatives, recognizing the potential harm of not doing so. Sometimes these were relatives with whom they had no, or a poor, relationship. Patients saw this task as more onerous and complex than simply “passing on” a letter. Previous research has framed this obligation to relatives as a kind of genetic responsibility (e.g., Hallowell et al. 2003). According to one systematic review, discharging responsibility towards family members and preventing illness are two of the four core functions that communicating within families about genetic risk serves. The other two functions are seeking emotional support and advice and obtaining information from the family (Wiseman et al. 2010). This same systematic review showed that patients believed that they were responsible for disseminating, or felt obliged to disseminate, genetic information. Similarly, participants in our study were tasked not only with coping with their diagnosis or test result but also with managing the consequences of telling relatives and questions of how much they should encourage relatives to have a test. In a recent discussion-paper, illustrated with real life cases, Mendes et al. (2017) highlight that patients navigating these same questions can sometimes exert pressure on family members to have a test out of a concern for their welfare. This can threaten these family members’ autonomous decision-making processes for whether to have a genetic test, which in turn, can increase the risk of family conflict. HCPs in previous studies have expressed concerns that patients might exert pressure on relatives to have a test if communication is left up to them (Stol et al. 2010). Like Mendes et al. (2017), we found that handing out letters could lead to family tensions—and this undermines some of HCPs’ arguments against contacting at-risk relatives themselves, for example, that patients often have a relationship with relatives that makes them better placed than HCPs to communicate (Dheensa et al. 2015, 2017; Stol et al. 2010).

Although HCPs aim was for the letters to benefit relatives, they were rarely aware of whether and to whom letters had been passed; and sometimes a letter, and thereby the dissemination of information, was “blocked” by family members or shared inappropriately. So-called blockers are apparently common: one study with 183 women with BRCA1/2 mutations showed that on average, each participant had one blocker in their family, tending to be the participants’ male spouses/partners and first-degree relatives (Koehly et al. 2009).

It is clear from these findings and previous research that patients might need support when talking to relatives about risk. However, as Mendes et al. (2016) point out, HCPs sometimes offer only information-based help—aiding patients’ understanding of their risk or condition—that focuses on the individual, rather than the family unit. HCPs also indicate they do not always address issues of family communication in their practice (Forrest et al. 2010).

### What Might Be some Useful Supplements to Using Family Letters?

It is worth considering some alternative and supplementary approaches to communication, which HCPs might discuss with patients to determine which they prefer. For example, HCPs might send letters directly to patients, which intervention research has shown can lead to an increase in testing uptake among at-risk relatives (Evans et al. 2009; Suthers et al. 2006). Patients who want help would then be relieved of the burden of communicating and HCPs would be able to track whether relatives had been contacted. These intervention studies are limited because testing uptake is not necessarily the best marker of successful communication. Relatives might still decide not to respond to letters from HCPs —they might decide not to seek testing, and letters might be frightening or too vague to inspire action. Indeed, a substantial proportion of relatives in the interventions that involved direct contact did not respond to letters, and many did not have a genetic test. For example, in Suthers et al.’s (2006) intervention cohort, on average only 40% of eligible relatives who had received letters from HCPs had their genetic status confirmed after two years.

An alternative to using letters is for patients or HCPs to contact at-risk relatives via web-based platforms, such as Kintalk.org, which could be set up, with the patient’s permission, to allow HCPs to contact relatives directly and/or to see whether relatives have responded to the information (Myers et al. 2013). “My Medical Record”, an electronic health record launched in several UK National Health Service trusts, has the potential to be developed in this way. Integrating such a tool into genetic practices would align with the broader shift towards digitization and integration of apps in healthcare services, but could widen inequality between patients from higher and lower socioeconomic status groups (Tudor-Hart 1971). There are also implications for patient confidentiality (e.g., if someone could access a patient’s record without permission) (Wynia and Dunn 2010). Many patients would still feel obliged to talk to their relatives about the risk even if the message was sent by a HCP or through an online tool. Thus, there would still be the need for support in this regard.

### Practice Implications

Based on these findings, we recommend that HCPs:

- help patients understand who should get the letters and why, e.g., using the family pedigree.
- discuss with patients about how they would like communication to proceed and to consider alternatives and supplements to standard practice.
- design letters with patient groups so that they are of an appropriate reading age and strike the balance between encouraging relatives to seek a referral without being overly directive or alarming.
- consider making direct contact with relatives/their GP as an alternative, or a supplement, to patients passing letters on.
- make it easier for patients who want help with communication to recontact HCPs.

It is especially important to improve communication practices as healthcare services incorporate broad genome tests, because these can produce a range of findings, including incidental findings, which can be relevant to patients’ relatives. Healthcare services plan to incorporate these tests into routine practice, meaning an increase in the number of patients and at-risk relatives is imminent. These tests will be ordered more frequently by “mainstream” specialties for whom such issues will be new territory, and who might need to liaise with clinical genetic services. Most HCPs in our study had genetics training and were thus well placed to assist families with communication: further consideration is required as non-genetic HCPs become more involved in ordering tests. Given all of this, our work is continuing to see how we can develop My Medical Record to improve familial communication.

### Study Limitations

This study is the first to investigate the purpose, advantages, and drawbacks of using family letters. Our study was limited to the UK. This was for specific reasons: the country has a national health service and a well-established genetic service, where European recommendations guide practice about family letters. We nevertheless call for research about practices elsewhere.

Since this study did not originally set out to investigate the use of letters specifically, some background data, which would have been useful to contextualize our results, was missing—for example, how many patients gave out or received letters and level of experience of HCPs (as a proxy for how experienced they were at writing and issuing letters). We did not explore the issues around family letters according to different conditions—further research on this question could be useful.

As is common in qualitative research, a limitation is that our sample was self-selected and thus potentially biased towards people especially engaged with managing and discussing their condition. This might explain why none had refused to tell family members about their condition. As Robinson (2014) points out, “self-selection bias is not possible to circumvent in interview-based research, as voluntary participation is central to ethical good practice, therefore all a researcher can do is be aware of the possibility for bias and consider its possible impact on findings and generalizability” (p.36).

## Conclusion

Several studies have explored the process of communication about genetic risk but none has explored the use of family letters. In this study, participants perceived letters to fulfill several roles but their journey from HCP, to patient, to relative was problematic. HCPs struggled to write letters in a way that was easy to understand while informative and reassuring, and encouraging without being overly directive. The letters helped patients in that they could refer relatives to its contents for a better explanation about the risk. However, communication was still fraught with difficulties and the letters did little to ameliorate these. What’s more, letters could be shared too widely, leading patients to lose control over the information. HCPs realized that letters could symbolize “finality” to patients—that the clinical encounter was over—and as such, HCPs thought that patients needed more support talking to relatives. We recommend that HCPs talk to patients about alternative and supplementary ways of communicating with relatives; that direct contact might be more appropriate in some cases; and that extra support is merited. These issues are pressing because mainstream specialties are now feeding back primary and unexpected incidental findings from broad genome tests. Patients will need support with talking to relatives about ever-more complex information. Thus, the next stage of our work will involve the development of an online resource to facilitate communication.
